# ANTP‐SmacN7 fusion peptide‐induced radiosensitization in A549 cells and its potential mechanisms

**DOI:** 10.1111/1759-7714.13393

**Published:** 2020-03-10

**Authors:** Rongxin Zhang, Hao Sun, Hong Wang, Wenxue Zhang, Kai Geng, Qiang Liu, Ping Wang

**Affiliations:** ^1^ National Clinical Research Center for Cancer, Key Laboratory of Cancer Prevention and Therapy, Tianjin's Clinical Research Center for Cancer Tianjin Medical University Cancer Institute and Hospital Tianjin China; ^2^ Radiotherapy Department Tianjin Medical University General Hospital Tianjin China; ^3^ Tianjin Key Laboratory of Radiation Medicine and Molecular Nuclear Medicine Chinese Academy of Medical Sciences & Peking Union Medical College Institute of Radiation Medicine Tianjin China; ^4^ Chinese Academy of Medical Sciences & Peking Union Medical College Institute of Biomedical Engineering Geriatric Health Engineering Research Center Tianjin China

**Keywords:** A549 cell line, apoptosiscaspase activationradiation sensitizationSmac

## Abstract

**Background:**

Radioresistance in tumors limits the curative effect of the radiotherapy. Mimetic compounds of second mitochondria‐derived activator of caspase (Smac) are potential new tumor radiation‐sensitizing drugs because they can increase radiation‐induced tumor cell apoptosis. Here, we observed the radiosensitization effect of a new Smac mimetic Antennapedia protein (ANTP)‐SmacN7 fusion peptide in A549 cells and investigated the underlying mechanisms behind the effects of this protein on tumor cells.

**Methods:**

The ANTP‐SmacN7 fusion peptide was synthesized and linked with fluorescein isothiocyanate to observe the protein's ability to penetrate cells. A549 cells were divided into the control, radiation‐only, ANTP‐SmacN7‐only and ANTP‐SmacN7 + radiation groups. The cells were exposed to 0, 2, 4 and 6 Gy, with 20 μmol/L of ANTP‐SmacN7. The radiation‐sensitizing effects of the ANTP‐SmacN7 fusion proteins were observed via clonogenic assay. Apoptosis was detected using flow cytometry. A comet assay was used to assess DNA damage. The levels and degrees of cytochrome‐c, PARP, H2AX, caspase‐8, caspase‐3, and caspase‐9 activation were detected via western blot assay. The radiation sensitization of the fusion peptide, expression of γ‐H2AX and C‐PARP were compared after adding the caspase inhibitor, Z‐VAD.

**Results:**

ANTP‐SmacN7 fusion proteins entered the cells and promoted A549 cell radiosensitization. Treatment with ANTP‐SmacN7 + radiation significantly reduced the A549 cell clone‐forming rate, increased the cytochrome‐c, cleaved caspase‐8, cleaved caspase‐3 and cleaved caspase‐9 expression levels, promoted caspase activation, and increased the rate of radiation‐induced apoptosis. The ANTP‐SmacN7 fusion peptide significantly increased radiation‐induced double‐stranded DNA rupture in the A549 cells and increased DNA damage. Adding Z‐VAD reduced the fusion peptide's proapoptotic effect but not the level of double‐stranded DNA breakage.

**Conclusions:**

The ANTP‐SmacN7 fusion peptide exerted a remarkable radiosensitization effect on A549 cells. This protein may reduce tumor cell radioresistance by inducing caspase activation and may be a potential new Smac mimetic that can be applied in radiosensitization therapy.

## Introduction

The role and position of radiotherapy in tumor therapy has become increasingly prominent. Radiotherapy is one of the main methods of treating malignant tumors. However, radioresistance of tumor cells during radiotherapy is the main problem limiting the efficacy of radiotherapy. Therefore, studying radiosensitization in tumor cells has become a research focus in recent years. Inhibitors of apoptosis proteins (IAPs) are overexpressed in numerous cancers and are associated with radiotherapeutic resistance in cancer cells.^1^ The second mitochondria‐derived activator of caspase (Smac) is an apoptosis‐promoting protein,^2^, ^3^ which, when affected by apoptosis‐inducing factors (eg, anticancer drugs, ionizing radiation, ultraviolet rays, chemical signals and DNA damage), can be released into the cytoplasm through the mitochondrial membrane with other mitochondrial proteins, such as cytochrome‐c, which is involved in regulating apoptosis by eliminating IAPs.^4^ The seven amino acid residues, SmacN7, at the amino terminus of Smac is the smallest active unit of the Smac protein. It can increase radiosensitization by inhibiting the effect of IAPs; however, SmacN7 cannot enter cells.^5^ Research shows that the third α‐helix (the 16 amino acid residues between 43 and 58) of the homeodomain of the Drosophila Antennapedia protein (ANTP) is the smallest protein region with transduction functions, which is independent of the receptor, channel, energy and endocytosis. ANTP can act directly on the lipid bilayer to complete transmembrane movement and enter cells.^6^ Therefore, we used the ANTP protein as the guiding peptide to synthesize the ANTP‐SmacN7 fusion peptide and guide SmacN7 into cells to radiosensitize the cells.

## Methods

### Materials and main reagents

The lung cancer cell line, A549, was preserved in the Radiation Injury Effect Laboratory of the Institute of Radiology. Trypsin and ethylenediamine tetraacetic acid (EDTA) lysate, 10% fetal bovine serum (FBS), and RPMI 1640 culture medium were purchased from Gibco (US). Phosphate‐buffered saline (PBS) was prepared at the Radiation Injury Effect Room of the Institute of Radiology. The positive fluorescence microscope was purchased from Nikon (Japan), and the flow cytometry equipment was purchased from Beckman Company (US).

### Peptide synthesis and cell penetration testing of the ANTP‐SmacN7 fusion peptide

To determine whether the ANTP‐SmacN7 protein could enter tumor cells, the C‐terminus of the fusion peptide was labeled with fluorescein isothiocyanate (FITC). The ANTP, SmacN7, and ANTP‐SmacN7 recombinant proteins were synthesized by Shanghai Sangon Biotech Co. Ltd., and contained the following amino acid sequences:

ANTP: Arg‐Gln‐Ile‐Lys‐Ile‐Trp‐Phe‐Gln‐Asn‐Arg‐Arg‐Met‐Lys‐Trp‐Lys‐Lys‐FITC;

SmacN7: Ala‐Val‐Pro‐Ile‐Ala‐Gln‐Lys‐Pro‐FITC;

ANTP‐SmacN7:Ala‐Val‐Pro‐Ile‐Ala‐Gln‐Lys‐Pro‐Arg‐Gln‐Ile‐Lys‐Ile‐Trp‐Phe‐Gln‐Asn‐Arg‐Arg‐Met‐Lys‐Trp‐Lys‐Lys‐FITC.

Cells were cultured for 3 h with 20 μmol/L of ANTP‐SmacN7, then entry of the fusion peptide into the cells was observed via fluorescence microscopy.

### Clone‐formation experiment

Each well of a 6 well plate was seeded with 400 cells for 24 hours, then the prepared drug solution was added for 24 hours. The concentration of the corresponding drug was 20 μmol/L. The cells were subsequently irradiated in room temperature air using a 137 Cs γ source (CAMMA‐CELL40; Atomic Energy of Canada Limited, Chalk River, ON, Canada). The source target distance was 30 cm, and the dose rate was 0.793 Gy/minute. After irradiation, cell cultures were grown at 37°C and terminated after 2–3 days. Cells were fixed and stained, and the experiment was repeated three times. At least 50 cells were defined as a clone colony. The clone‐formation rate (%) = the number of clone colonies/the number of inoculated cells × 100%. A cell survival curve was fitted based on a single‐hit multitarget equation, and the curve parameter D0 value was calculated. The radiosensitization ratio (SER) = the D0 of the irradiation group/the D0 of the dosed irradiation group.

### Apoptosis detected by Annexin V and PI double staining method

Cells were harvested and digested with EDTA‐free trypsin, then suspended in 500 μL of binding buffer with 5 μL Annexin V‐FITC. The suspension was mixed evenly. A total of 5 μL of propidium iodide was added and mixed evenly. The cell suspension was protected from light and incubated at room temperature for about 5–15 minutes. Fluorescence was detected by flow cytometry in an hour (excitation wavelength *Ex* = 488 nm; emission wavelength *Em* = 530 nm). Green fluorescence of Annexin V‐FITC was detected by FITC channel (FL1); PI red fluorescence was detected by PI channel (FL2/3).

### Western blot

Cells were collected when they reached a subfused state and lysed with MER‐Pierce lysate. Proteins were quantified as per the BCA Protein Assay Kit instructions (Takara, Shiga, Japan). A total of 50 μg of proteins were used for discontinuous polyacrylamide gel electrophoresis, electrotransferred to a polyvinylidene difluoride membrane and blocked with TBST containing 5% skim milk at 4°C overnight. The membranes were incubated with primary antibodies at room temperature for 2 hours, then washed, incubated with the corresponding horseradish peroxidase‐conjugated secondary antibody at room temperature for 1 hour, and detected by ECL chemiluminescence. β‐actin was used as an internal reference.

### Comet assay

Cells in the logarithmic growth stage were inoculated into 6 well plates at 2 × 10^5^ cells per well. After the cells were adhered to the plates, single cell suspensions were prepared 24 h after irradiating the slides with 0 Gy or 4 Gy γ‐rays. Normal melting‐point agarose gel was spread on the glass slides. After cooling, the cell suspension was fully mixed with low melting‐point agarose gel, then spread on the normal melting‐point agarose gel. The slides were immersed in cell lysate and cracked at 4°C for 2.5 hours. The slides were rinsed with PBS, then transferred to the electrophoresis solution at 4°C for 20 minutes, followed by electrophoresis at 30 V for 20 minutes. The slides were then placed in neutralization buffer for 20 minutes, rinsed with PBS and stained with ethidium bromide (2 μg/mL) for approximately 20 seconds. The slides were observed and photographed under fluorescence microscopy. Comet images of at least 100 cells were randomly captured and analyzed using CASP software. Each experiment was repeated at least three times.

### Statistical analysis

Analysis was conducted using SPSS 17.0 statistical software. Each experiment was repeated at least three times independently. Data are presented as the mean ± SEM. Pairwise comparisons were conducted using *t*‐test. Differences among groups were analyzed using chi‐square tests. ELSURrad98 software was used to calculate the drug sensitization ratio and plot the cell survival curve. A cutoff of *P* < 0.05 was considered statistically significant.

## Results

### ANTP‐SmacN7 fusion peptide entered the cells

**Figure 1:**
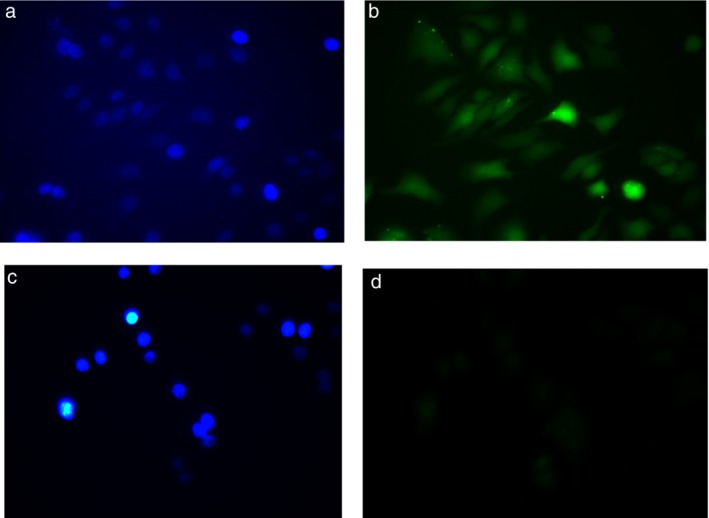
Comparison of cell transduction capabilities. (**a**), (**b**) FITC‐ANTP‐SmacN7 group (ANTP‐SmacN7 fusion peptide entered the cells); (**c**), (**d**) FITC‐SmacN7 group (fusion peptide not entered the cells). DAPI‐stained (blue) cells are living cells, FITC‐stained (green) cells show that the peptide entered the tumor cells.

Cells were collected and the cell concentration was adjusted to 5–10 × 10^4^ cells/mL. Then, 24 hours after cell adherence, cells were cultured for 3 h with 20 μmol/L of ANTP‐SmacN7 or SmacN7, and entry of the fusion peptide into the cells was observed via fluorescence microscopy. The control group was stained with DAPI solution to determine the location of the nucleus. The ANTP‐SmacN7 fusion protein successfully entered and accumulated in the cells, enabling the proapoptotic effect of the fusion peptide in the cells (Fig 1).

### Radiosensitization effect of ANTP‐SmacN7 on A549 cells

**Figure 2:**
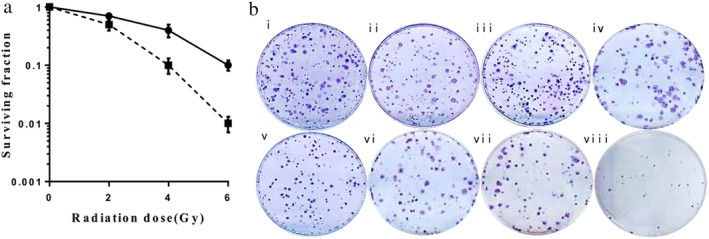
ANTP‐SmacN7 increased the radiosensitivity of A549 cells. (**a**) Survival fraction of the irradiation and irradiation+ANTP‐SmacN7 group. (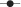) control (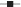) ANTP‐SmacN7. (**b**) Clone‐forming assay: (**i**) control group; (**ii**) ANTP group; (**iii**) SmacN7 group; (**iv**) ANTP‐SmacN7 group; (**v**) Irradiation group; (**vi**) Irradiation + ANTP group; (**vii**) Irradiation + SmacN7 group; (**viii**) Irradiation + ANTP‐SmacN7 group.

A549 cells in the logarithmic growth phase were incubated with ANTP, SmacN7 and ANTP‐SmacN7, respectively. Cells in the irradiation group were irradiated with 0, 2, 4, and 6 Gy irradiation. The irradiation combined with ANTP‐SmacN7 group received 20 μmol/L of ANTP‐SmacN7 for 24 hours. The cells were subsequently irradiated. After irradiation, cell cultures were grown at 37°C and terminated after three days. At least 50 cells were defined as a clone colony. A cell survival curve was fitted based on a single‐hit multitarget equation. The clone‐formation rate (%) = the number of clone colonies/the number of inoculated cells × 100%. Experiment was repeated three times independently. As shown in Fig 2, ANTP‐SmacN7 enhanced the radiosensitivity of the A549 cells, compared to the irradiation group. The clone‐formation rate was significantly decreased in the irradiation+ANTP‐SmacN7 group. The irradiation‐only, ANTP‐SmacN7‐only and ANTP‐SmacN7 + irradiation groups differed significantly (*P* < 0.01; *P* < 0.01). The ANTP‐SmacN7 fusion peptide exerted a radiosensitizing effect when combined with irradiation (Fig 2).

### Proapoptotic effect of the ANTP‐SmacN7 fusion peptide

**Figure 3:**
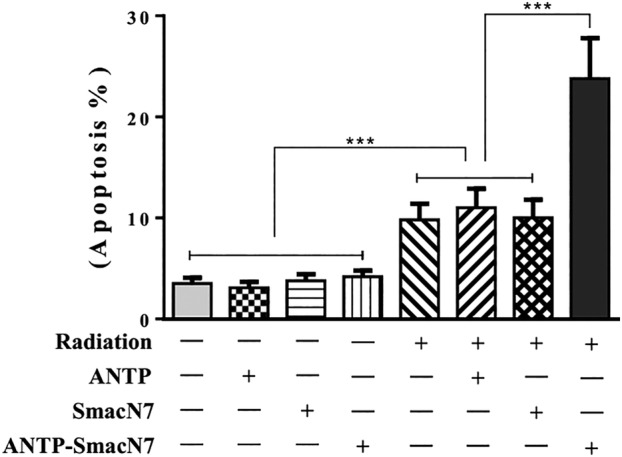
Effect of ANTP‐SmacN7 on apoptosis in A549 cells. Apoptosis of different groups (^***^ *P* < 0.01).

To observe the effects of ANTP‐SmacN7 fusion proteins on radiation‐induced apoptosis, 20 μmol/L ANTP, SmacN7 and ANTP‐SmacN7 were incubated with A549 cells, respectively. Cells in combination groups were irradiated with γ‐rays (4 Gy). Cells were harvested 48 hours after irradiation. Apoptosis was detected using Annexin V‐FITC and PI double staining. Calculated the mean ± SEM. The experiment was repeated three times. Figure 3 shows the apoptosis levels of the A549 cells in each group. The apoptotic rate of the combined group differed significantly from the other groups (*P* < 0.01). ANTP‐SmacN7 alone did not promote apoptosis, but in the combined group, ANTP‐SmacN7 significantly increased apoptosis in the irradiated cells, further confirming that ANTP‐SmacN7 has a radiosensitizing effect.

### Comet analysis enabled quantitatively detecting radiation‐induced DNA damage in A549 cells induced with the ANTP‐SmacN7 fusion peptide

**Figure 4:**
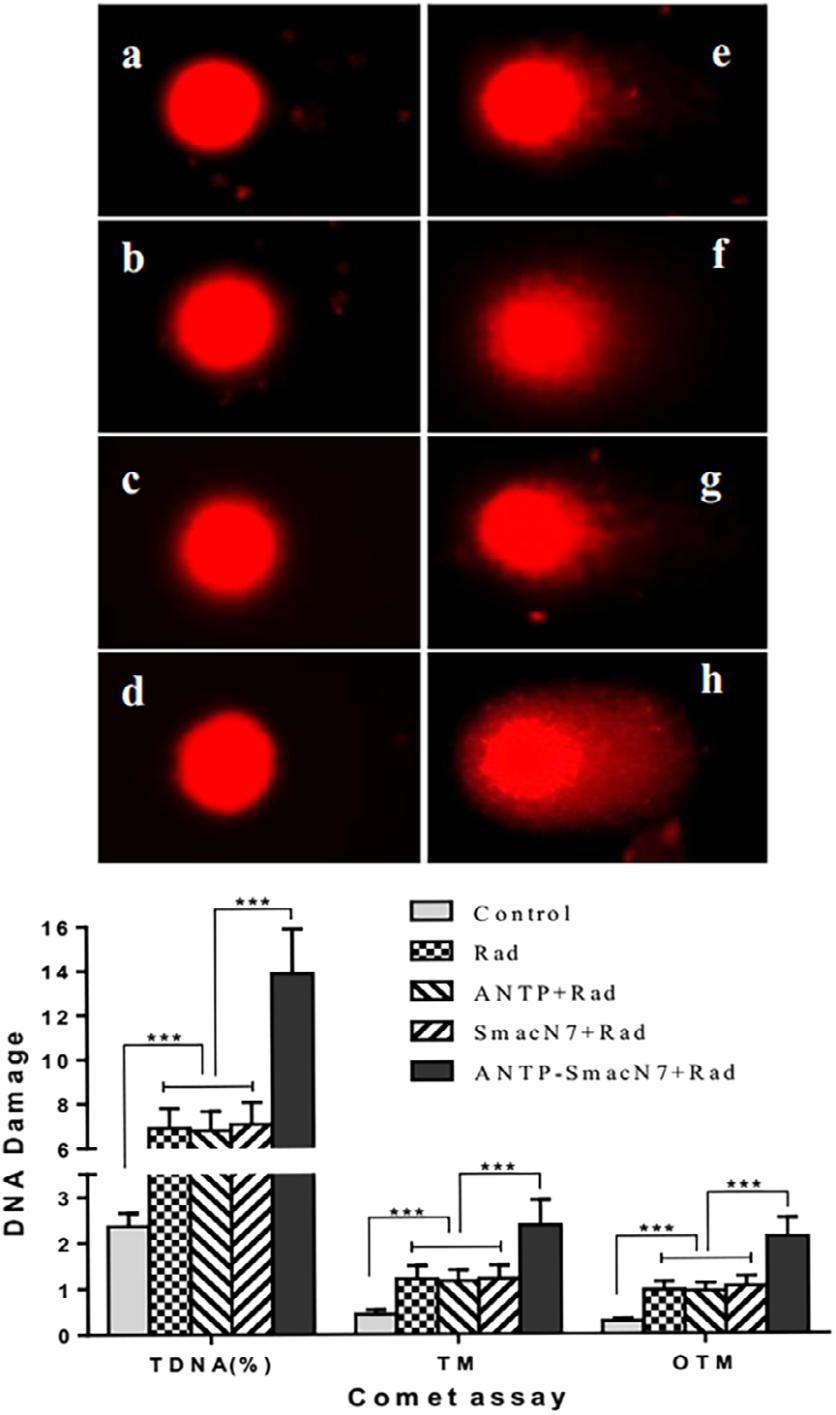
Comet analysis enabled detecting radiation‐induced DNA damage in A549 cells. (**a**) Control group; (**b**) ANTP group; (**c**) SmacN7 group; (**d**) ANTP‐SmacN7 group; (**e**) Irradiation group; (**f**) Irradiation+ANTP group; (**g**) Irradiation+SmacN7 group; (**h**) Irradiation+ANTP‐SmacN7 group (^***^ *P* < 0.01). (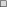) Control (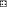) Rad (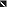) ANTP+Rad (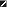) SmacN7+Rad (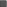) ANTP‐SmacN7+Rad.

To observe the effects of ANTP‐SmacN7 fusion proteins on radiation‐induced DNA damage, 20 μmol/L ANTP, SmacN7 and ANTP‐SmacN7 were incubated with A549 cells, respectively. After 12 hours, cells in combination groups were irradiated with γ‐rays (4 Gy). Cells were collected 48 hours after irradiation. The comet assay detected the degree of DNA damage, with tail DNA% (TDNA %), tail moment (TM) and Olive tail moment (OTM) as evaluation and analysis indicators. Comet images of at least 100 cells were randomly captured and analyzed using CASP software. Data were presented as the mean ± SEM; each experiment was repeated at least three times. As the TDNA%, TM and OTM increase, the severity of the radiation damage increases. The TDNA%, TM and OTM were significantly higher in the irradiation+ANTP‐SmacN7 fusion peptide group than in all other groups (all *P* < 0.05; Fig 4), indicating that the ANTP‐SmacN7 fusion peptide significantly enhanced the DNA damage induced by ionizing irradiation.

### Western blot detected caspase activation and PARP of A549 cells in the different groups

**Figure 5:**
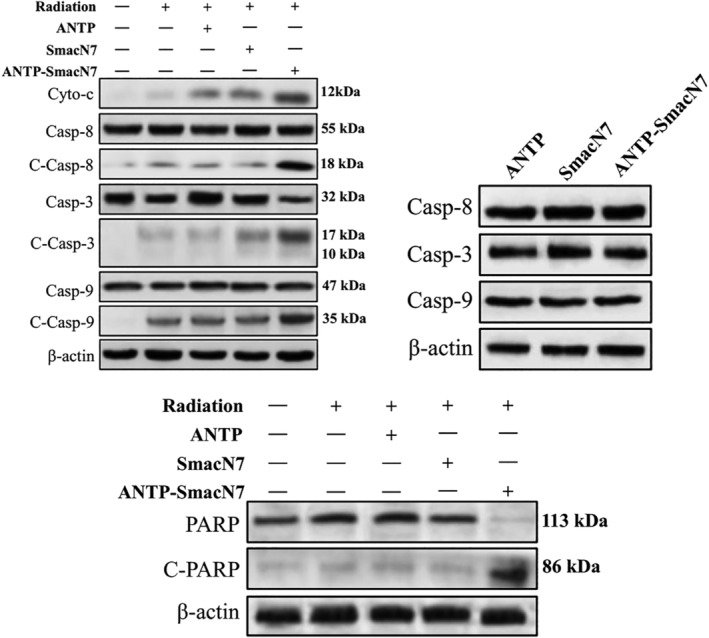
Caspase and PARP expressions in the cells of each experimental group.

A549 cells were incubated with 20 μmol/L ANTP, SmacN7 and ANTP‐SmacN7, respectively. After 12 hours, cells were irradiated with γ‐rays (0 or 4Gy), according to experimental design. Cells were harvested 48 hours after irradiation. Changes in caspase‐3, 8, 9, PARP and cleaved caspase‐3, 8, 9, cleaved PARP were detected by western blot with or without ANTP‐SmacN7 effect. Each experiment was repeated three times. The results demonstrated that the ANTP‐SmacN7 fusion peptide group promoted the expression of cytochrome, as well as cleavage of caspases and PARP in combination with radiotherapy, compared with ANTP, SmacN7 group. ANTP‐SmacN7 fusion peptides promoted caspase activation in the radiation‐induced apoptosis of A549 cells (Fig 5).

### Addition of caspase inhibitor, Z‐VAD, weakened the radiosensitizing effect of the fusion peptide on A549 cells

**Figure 6:**
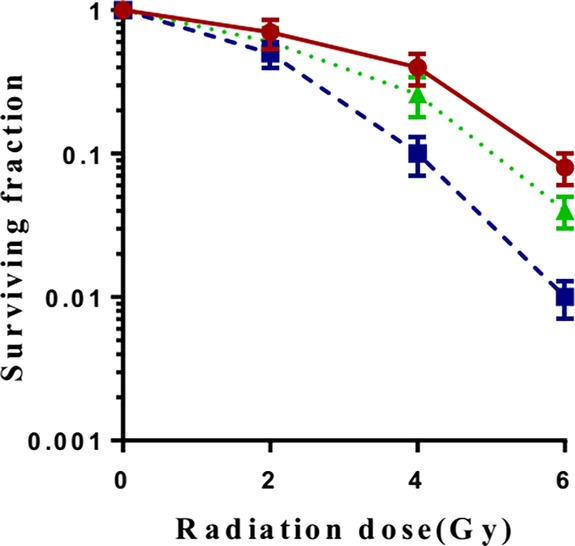
Caspase inhibitor Z‐VAD weakened the radiation sensitization of the fusion peptide. (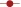) control (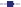) ANTP‐SmacN7 (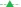) z‐VAD.

The caspase inhibitor, Z‐VAD‐FMK, is a cell‐permeable pan‐caspase inhibitor that penetrates cell membranes and inhibits apoptosis due to caspase activation. A549 cells in the logarithmic growth phase were divided into the control group, the ANTP‐SmacN7 group and Z‐VAD combined with ANTP‐SmacN7 group, each group with two repeated culture holes. The concentration of z‐VAD was 10 μmol/L, while ANTP‐SmacN7 was 20 μmol/L. Z‐VAD and ANTP‐SmacN7 were incubated with A549 cells, respectively. After 4 hours, cells were irradiated with γ‐rays at different doses (0, 2, 4, 6 Gy). Cells were harvested 48 hours after irradiation. Cells were fixed and stained, and the experiment was repeated three times. At least 50 cells were defined as a clone colony. The clone‐formation rate (%) = the number of clone colonies/the number of inoculated cells × 100%. A cell survival curve was fitted based on a single‐hit multitarget equation. Figure 6 shows that administering Z‐VAD weakened the radiation sensitization of the fusion peptide, suggesting that the radiation sensitization of the ANTP‐SmacN7 fusion peptide on A549 cells was caspase‐dependent.

### Effects of the ANTP‐SmacN7 fusion peptide and Z‐VAD on double‐stranded DNA ruptures in A549 cells

**Figure 7:**
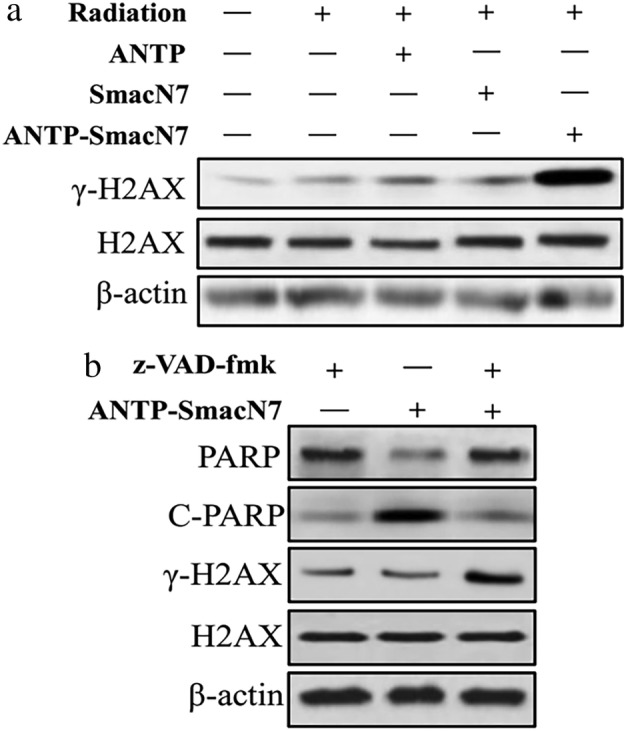
(**a**) Protein expression of γ‐H2AX and H2AX in different groups; (**b**) Protein expression of PARP, C‐PARP, γ‐H2AX and H2AX in the Z‐VAD and ANTP‐SmacN7 fusion peptide groups.

A total of 549 cells in the logarithmic growth phase were divided into the control, ANTP, SmacN7, the ANTP‐SmacN7, Z‐VAD and Z‐VAD combined with ANTP‐SmacN7 groups. A549 cells were incubated with Z‐VAD, ANTP, SmacN7 and ANTP‐SmacN7, respectively. The concentration of z‐VAD was 10 μmol/L, while ANTP, SmacN7, ANTP‐SmacN7 was 20 μmol/L. After 12 hours, cells were respectively irradiated with γ‐rays (0 or 4 Gy). Cells were harvested 48 hours after irradiation. Changes in γ‐H2AX and H2AX of different groups were detected by western blot. Changes in PARP and c‐PARP of ANTP‐SmacN7 group, Z‐VAD group and Z‐VAD combined with ANTP‐SmacN7 group, were detected by western blot. Each experiment was repeated three times. The research shows that The ANTP‐SmacN7 fusion peptide significantly increased the irradiation‐induced double‐stranded DNA breaks in the A549 cells, and administering the caspase inhibitor reduced apoptosis but not the double‐stranded DNA breaks (Fig 7).

## Discussion

Because of the high prevalence and mortality of lung cancer, it is considered a major public health problem worldwide. Studying the pathogenesis and treatment tolerance mechanisms of lung cancer is a key issue in treating lung cancer. Evasion and dysregulation of apoptosis are the essential hallmarks of human cancer cells and represent important bases of resistance to current treatment approaches, including radiation.^7^, ^8^

Evidence indicates that IAPs are frequently overexpressed in various cancers, and their expression levels are implicated in tumorigenesis, tumor progression, treatment resistance, treatment failure, and poor prognoses.^9^, ^10^, ^11^, ^12^, ^13^, ^14^ IAPs were originally discovered in baculoviral genomes by Miller and colleagues in 1993. IAPs comprise a family of antiapoptotic proteins that promote prosurvival signaling pathways and prevent apoptosis by interfering with caspase activation.^15^ Smac is an endogenous functional protein that inhibits IAPs^16^ and promotes apoptosis by activating caspases along with cytochrome‐c. Smac is normally a mitochondrial protein but is released into the cytosol when cells undergo apoptosis. Mitochondrial importation and cleavage of its signal peptide are required for Smac to gain its apoptotic activity. IAP function can be regulated and suppressed by binding to Smac derived from mitochondria following proapoptotic signaling. Smac overexpression increases cells' sensitivity to apoptotic stimuli.^17^ IAP inhibition has attracted extensive attention over the last decade. With the in‐depth study of IAP functional sites, the antitumor and radiosensitizing effects of Smac and its mimetics have become an important research focus.^18^, ^19^, ^20^, ^21^, ^22^, ^23^, ^24^, ^25^, ^26^, ^27^, ^28^, ^29^

This study was based on previous experimental results that identified the radiosensitizing activity of small molecule SmacN7, which contains an apoptosis‐promoting sequence that can destroy IAP binding to caspase‐9 and caspase‐3. ANTP‐SmacN7 effectively improved cellular radiosensitivity.^30^, ^31^ Our study showed that ANTP‐SmacN7, as an IAP inhibitor, had a significant radiation‐sensitizing effect in A549 cells, indicating that ANTP‐SmacN7 could be used as an effective radiation sensitizer. This study focused on promoting apoptosis by transduction of exogenous Smac protein mimics into cells. To ensure both protein activity and cell penetration of Smac,^32^, ^33^ an ANTP transmembrane sequence was ligated to form a fusion peptide using the SmacN7 sequence. Our results also confirmed that ANTP‐SmacN7 penetrated the cell membrane and entered the cell after coculturing. Although the radiation‐sensitizing effect of ANTP‐SmacN7 alone on A549 cells was minimal, combining radiation with ANTP‐SmacN7 significantly promoted radiation‐induced apoptosis. IAPs prevented caspase activation by blocking caspase cleavage and activation. However, release of Smac blocked the IAPs, allowing downstream caspase activation.

In this study, the expression levels of cytochrome‐c, caspase‐3, −8, and − 9, c‐caspase‐3, −8, and − 9, and PARP proteins were detected in A549 lung cancer cells. The ANTP‐SmacN7 fusion peptide promoted caspase activation in radiation‐induced apoptosis, which enhanced A549 cell radiosensitivity. Z‐VAD‐FMK, a broad‐spectrum caspase inhibitor, was used as an antiapoptotic drug to prevent apoptosis.^34^ These results confirmed that the radiation sensitization of the ANTP‐SmacN7 fusion peptide to A549 cells depended on apoptotic caspase.

DNA damage is a critical lethal factor after cells are exposed to ionizing radiation and can be used as a reference index for radiosensitivity.^35^ In this study, a comet assay quantitatively detected ANTP‐SmacN7 fusion peptide‐induced radiation DNA damage in A549 cells. The TDNA%, TM and OTM of the radiation+ANTP‐SmacN7 fusion peptide group were higher than those of the other groups, indicating more severe DNA damage. Measurement of γ‐H2AX focal levels in cells is a sensitive and reliable method for quantifying the radiation‐induced DNA damage response.^36^ In this study, the ANTP‐SmacN7 fusion peptide combined with irradiation significantly increased the radiation‐induced double‐stranded DNA breaks in A549 cells, and adding a caspase inhibitor reduced apoptosis but not the double‐stranded DNA breaks.

In conclusion, this study confirmed that ANTP‐SmacN7 is an effective radiation sensitizer, providing a scientific theoretical basis for ANTP‐SmacN7 as a novel tumor radiotherapy sensitizer. ANTP‐SmacN7 exhibits promising application prospects for treating cancer. Further animal experiments and preclinical studies are needed before large‐scale application, which should be further explored in future work.

## Disclosure

The authors declare there are no conflicts of interest.
